# Student-run free clinics may enhance medical students’ self-confidence in their clinical skills and preparedness for clerkships

**DOI:** 10.1080/10872981.2024.2348276

**Published:** 2024-05-02

**Authors:** Venina S. Kalistratova, Arina Nisanova, Lucy Z. Shi

**Affiliations:** a School of Medicine, University of California Davis, Davis, CA, USA; b Department of Internal Medicine, University of California Davis, Davis, CA, USA

**Keywords:** Student-run free clinics, medical education, learning, clinical skills, interprofessional education

## Abstract

**Introduction:**

Student-run free clinics (SRFCs) offer medical students a unique opportunity to develop their clinical, diagnostic, and social skills while providing care to medically underserved communities. This study aims to evaluate the value of SRFC involvement on students’ self-reported confidence in various clinical domains and satisfaction with their medical education.

**Methods:**

We conducted a single-center retrospective pre-post assessment at an urban academic institution among second- to fourth-year medical students. We administered a 25-item questionnaire capturing the scope of clinic involvement and assessing self-reported confidence in multiple clinical domains following a one-year-long participation in student-run free clinics.

**Results:**

Fifty-six students completed the survey. Participation in SRFCs significantly increased self-reported confidence in patient history-taking (*p* < 0.001), performing oral presentations (*p* < 0.001) and physical exams (*p* < 0.001). Students also reported significantly greater confidence in working with translators (*p* < 0.001) or as part of an interprofessional team (*p* < 0.001) and understanding the needs of the population served (*p* < 0.001). Students also found SRCs to significantly improve their confidence in preparedness for clerkships (*p* < 0.001). SRFC involvement can improve medical students’ confidence in their clinical and interpersonal skills and enhance preparedness for clerkships and working with diverse patient groups.

**Conclusion:**

SRFCs are a useful tool in the medical school curriculum that help bridge the gap between classroom learning and clinic and may encourage practice in medically underserved communities. SRFCs also integrate classroom material and clinical practice, although standardized evaluation metrics need to be developed. SRFCs should be incorporated as a learning experience by medical schools nationwide.

## Introduction

Student-run free clinics (SRFCs) are a staple of medical education across the United States and have been the training ground for generations of physicians and healthcare workers. SRFCs often operate in association with local medical schools and are staffed by volunteer medical students under the supervision of licensed physicians. It is estimated that up to 75% of medical schools have at least one associated student-run clinic [1]. They serve the dual purpose of providing care to traditionally medically underserved communities and offering medical students a unique opportunity to develop their clinical, diagnostic, and social skills [2].

Student-run clinics mainly serve patients of low socioeconomic status who often fall below the federal poverty line [3]. These clinics predominantly provide cost-free services to patients who require long-term management for chronic conditions, such as diabetes mellitus, hypertension, obesity, anxiety, or depression. Student-run clinics tend to operate on a low budget while providing a vast array of services, including both primary and specialized care as well as immigration consultations and lifestyle coaching [4]. A large part of the benefits derive from the reduction in the emergency department (ED) visits among uninsured patients seeking urgent care, especially those fluent in Spanish who rely on interpreters [5,6].

Medical students who participate in student-run clinics may derive a different set of benefits, such as enhanced clinical skills and empathy towards patients from different walks of life [7]. SFRCs involvement has been shown to facilitate leadership skills development and increase confidence in working with interprofessional teams and teaching others. SRFC participation may further help guide career choice and has been linked with an increased commitment to working with underserved communities [8]. However, the impact of SRFCs may be difficult to isolate from the broader context and contribution of the medical curriculum. We conducted a pre/post survey among medical students in their second to fourth year of training aimed to evaluate the value of SRFC involvement on students’ self-reported confidence in various clinical domains.

## Methods

This is a single-center retrospective pre-post assessment study that surveyed second through fourth-year medical students at the University of California Davis School of Medicine. Between September 2022 and January 2023, we contacted all enrolled medical students using email outreach and included a link to the survey. The 25-question survey was administered using the Qualtrics platform (Provo, UT). The survey captured sociodemographic data, the scope of clinic involvement, including roles, type of clinic and frequency of involvement, motivations for joining the clinic, and members of interdisciplinary teams the students interacted with. The survey also included 14 pre-post questions assessing students’ confidence in multiple clinical domains before and after a one-year-long involvement in student-run clinics. Students were asked to rate their confidence in taking patient history, performing a physical exam, performing an oral presentation, understanding the needs of the population served, working with an interpreter, working in interdisciplinary teams, and preparedness for clerkships on a 5-item Likert scale (not at all confident, slightly confident, somewhat confident, fairly confident, completely confident). Lastly, students were also asked to reflect on whether their Objective Structured Clinical Examination (OSCE) scores have improved, stayed the same, or worsened following involvement in clinics.

The data were analyzed and depicted using the Wilcoxon matched pairs signed rank test and the difference in differences analysis in GraphPad Prism 9 (San Diego, CA, version 9.5.0). The study was conducted in accordance with the Declaration of Helsinki. The Institutional Review Board approved the study protocol at the University of California, Davis (IRB: 1955540-1). All participants signed an informed consent document prior to enrollment.

## Results

Fifty-six medical students (10%) of the 539 students enrolled at the University of California, Davis School of Medicine took part in the study. Students were not compensated for participation. Participants’ characteristics, clinic roles, and motivations for SRFC involvement are summarized in Table 1.

**Table 1.** Participant demographic characteristics, training level, and the scope of student-run free clinic (SRFC) involvement (*n* = 56).

		*n* (%)
Demographics	Underrepresented in medicine	25 (44.6%)
	Fluent in a second language	48 (85.7%)
Level of Training	Second year	30 (53.6%)
	Third year	10 (17.9%)
	Fourth year	14 (25%)
	Research year	2 (3.6%)
Clinic Roles	Co-director	41 (73.2%)
	Officer	12 (21.4%)
	Volunteer	3 (5.4%)
	Secondary roles in addition to a co-director or officer	44 (78.6%)
Frequency of clinics attended per month	One	20 (35.7%)
	Two	31 (55.4%)
	Three	2 (3.6%)
	Variable days	3 (5.4%)
Motivations for joining SRFC	Serve the underserved community	54 (96.4%)
	Serve the unhoused community	22 (39.2%)
	Serve a particular ethnic population	28 (50%)
	Improve clinical skills	53 (94.6%)
	Improve diagnostic skills	39 (70%)
	Improve curriculum vitae	34 (60.7%)

**Figure 1.**
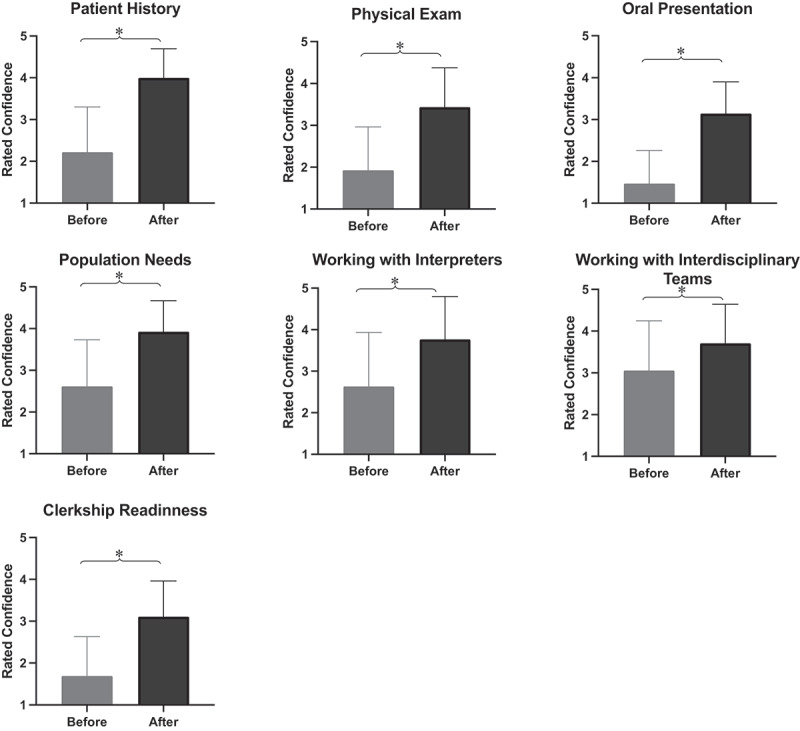
Medical students’ self-reported confidence in multiple clinical domains showing significantly increased confidence in various clinical skills and preparedness for clerkships following a one-year involvement in a student-run free clinic. Confidence was rated on a Likert scale (1 = not at all confident, 2= slightly confident, 3= somewhat confident, 4 = fairly confident, 5 = completely confident) using a Qualtrics survey.

Students mostly reported the same overall OSCE scores (36, 65.5%), 13 students noted improvement (23.6%) and 6 a decrease (10.9%) in scores following clinic involvement. Participation in SRCs significantly increased self-reported confidence in taking the patient history (slightly to fairly, *p* < 0.001), performing an oral presentation (not at all to somewhat, *p* < 0.001) and physical exam (slightly to somewhat, *p* < 0.001). Students also reported significantly greater confidence in working with translators (somewhat to fairly, *p* < 0.001) or as part of an interprofessional team (somewhat to fairly, *p* < 0.001), as well as confidence in understanding the needs of the population they served (somewhat to fairly, *p* < 0.001). Students also found SFRCs to significantly improve their confidence in preparedness for clerkships (not at all to somewhat, *p* < 0.001). Compared with officers, the scores of co-directors, who tend to have higher involvement in clinic, showed a greater average increase in confidence in all clinical domains with the exception of understanding the population’s needs, which was not a statistically significant finding. Notably, students who attended the clinic twice per month reported a significantly greater increase in confidence in their oral presentation skills (*p* < 0.05) and working with interpreters (*p* < 0.05) compared to those who participated once a month. The remaining improvements in confidence were significantly different based on the frequency of participation: students who attended SRFCs twice a month showed a greater average increase in confidence, including taking history, performing the physical exam, working in teams, and preparedness for clerkship. The change in confidence scores was not significantly different based on the students’ year in training for all survey domains except for the physical exam. Second-year students reported significantly greater improvement in the confidence of their physical exam skills compared to the third-year students (*p* < 0.05) (see Figure 1).

## Discussion

Student-run free clinics are organizations often linked to medical schools and universities that aim to provide care for medically underserved populations at no cost. The clinics are staffed by medical students and rely on the backing of local physicians. SRFC engagement is not mandatory, and students self-select to engage in this experience during the pre-clinical phase of the curriculum. Many clinics also benefit from other graduate students, including pharmacy, dental, psychology, social work, and law students. SRFCs provide a unique opportunity for students to enrich their education by interacting with patients early on in their careers as well as by working closely with mentoring physicians [9].

The reported benefits of student-run free clinics are manifold: they bridge the healthcare gap among uninsured patients by providing access to preventive care [10]. This has, in turn, resulted in the reduction of ED visits and saved thousands of dollars [4,5]. Many SRFCs have also expanded their scope of care to include subspecialties, such as gynecological or ophthalmological care, providing early detection and referrals for complex disease management [11–13]. Visits to SRFCs have also been linked with an increase in the use of reliable medical information [14].

Many studies have focused on the economic and social benefits of SRFCs but not on their contributions to medical education in the US [4–6]. This study surveyed students in one U.S. medical school about their own perception of the benefits derived from participating in SRFCs. The majority of those surveyed participated as co-directors of student-run clinics, a position that requires more extensive involvement, including clinic and supply management, scheduling, staffing, as well as care coordination. The larger scope of involvement, time commitment, and accountability required of co-directors likely explains why co-directors, on average, reported greater confidence in multiple domains than officers.

An additional benefit, and one which may help gain further understanding of the positive impact of SRFC service on student happiness and mental health, is the students’ motivation to join the clinics. The vast majority of students surveyed (54, 96.4%) reported their desire to serve traditionally underserved communities or currently unhoused patients (22, 39.3%). Half of the students surveyed reported their motivation to serve a particular ethnic community and expressed significantly greater confidence in understanding the needs of the specific community they served through SRFC involvement. This may result in more positive interactions between medical students and patients as well as an increase in patient satisfaction [15]. Thus, longitudinal involvement in SRFC may increase medical students’ commitment to cultural humility and help them develop tools to provide more inclusive and culturally sensitive care [16]. Exposure to diverse patient groups may further contribute to developing cultural humility and encourage students to practice in underserved areas and address the critical physician shortages in rural communities. The students surveyed also reported a greater confidence in understanding the needs of the specific communities they served during their time as volunteers in SRFCs [17].

Medical students volunteering at student-run free clinics may also derive the additional benefit of early exposure to working as part of an interdisciplinary team. The students surveyed reported significantly greater levels of confidence when working with interpreters after completing a year of volunteering in a clinic and a moderate increase in confidence when working in an interprofessional team. In recent years, interpreters have become an integral part of the U.S. healthcare system, serving a multi-ethnic population and bridging the cultural and linguistic gap between patient and provider [18]. Early exposure to working with interpreters may be essential to developing the skill of effective multilingual communication, which can further drive improved patients’ understanding of and decision-making capacity regarding their health.

Additionally, participants reported a significant increase in readiness to transition to the clerkship phase of their education. Despite the continuous evaluation of students’ clinical skills through OSCEs throughout the pre-clinical curriculum phase, the standardized encounters are limited in their ability to introduce students to a broad spectrum of medical conditions and lack the real-life feel. Early exposure to provider–patient interactions may help students develop their clinical skills faster and contextualize the basic science pre-clinical courses [19]. Clinical clerkship performance remains an important aspect of the Medical Student Performance Evaluation, which can be a tool to enhance candidates’ residency applications [20]. Therefore, introducing early clinical exposure that encompasses multiple pillars of clerkship evaluations, such as history taking, physical exam, and effectively synthesizing the obtained information into an oral presentation, may translate into an enhanced clerkship performance and benefit students’ careers and development as future providers. Early experience in various clinical specialties offered by SFRCs may additionally help guide the decision-making process in choosing the desired specialty [21].

Our study also highlights the lack of standardized metrics that assess the scope of involvement and the impact of student-run clinic participation on student learning and patient outcomes [1,22]. To date, several studies reported using individually developed questionnaires that focus on and investigate different aspects of SRFC involvement [5,7,9]. A systematic review of 92 SRFC-focused studies found a positive impact of clinics on clinical, interprofessional, and leadership skills, as well as developing empathy for the underserved population. However, the authors remarked on limitations in study quality and lack of perspective from outcomes from international medical schools [8]. Development of a standardized evaluation tool would enable institutions and clinics alike to assess the benefits and limitations of clinic involvement, facilitate informed quality improvement initiatives, and encourage more schools to establish SRFCs.

The limitations of this study included a relatively small sample and the retrospective nature of data collection. Only 10% of all enrolled students completed the survey, of which most served as clinic co-directors and officers, suggesting a potential selection bias. Our survey might have attracted students who felt more strongly of SRFCs, both positively and negatively. Additionally, our survey did not capture any students who did not participate in SRFCs, and thus we were unable to compare the confidence in clinical skills among clinical participants and those who did not have any clinic engagement. We also used a non-validated questionnaire to capture our findings. Another limitation of this study was using self-reported OSCE scores, thus relying on students’ honesty and willingness to disclose their performance results. We were unable to cross-reference these scores with the official student transcripts as we did not obtain FERPA (the Family Educational Rights and Privacy Act) consent. Considering the low baseline survey response rates, we did not pursue a longitudinal assessment of student experiences due to expected attrition and non-engagement in the follow-up surveys. A subsequent longitudinal prospective study might help confirm our findings and explore the impact of SRFC involvement to further mitigate recall bias inherent to a retrospective design of our study.

Despite these limitations, we captured significant effects of SRFC involvement on students’ confidence in multiple clinical domains. Additionally, our study highlights the lack of standardized metrics to assess medical students’ involvement in SRFCs. Medical schools should focus on developing standardized metrics for SRFC performance evaluation and its effect on clinical growth. Future studies should be designed as prospective and multi-institution to better capture the scope of benefits offered by SRFCs.

## Conclusion

SRFCs are an integral part of medical student training, providing clinical context for classroom learning, dedicated space to practice clinical skills, and increased cultural competency navigating the healthcare system, underserved communities, and interpreter services. They integrate early clinical exposure into the medical school curriculum and extend healthcare services to medically underserved communities. Our study indicates that SRFC involvement improves students’ confidence in their clinical and interpersonal skills and enhances preparedness for clerkships. Students were also more confident in understanding the needs of diverse communities, which may encourage students to practice with underserved populations and address provider shortages in rural communities. SRFCs can help bridge the gap between classroom learning and clinic. Standardized metrics need to be developed for meaningful, longitudinal assessment of benefits. We recommend that SRFCs be incorporated as a learning experience by medical schools nationwide.
